# INtimal hyPerplasia evAluated by oCT in de novo COROnary lesions treated by drug-eluting balloon and bare-metal stent (IN-PACT CORO): study protocol for a randomized controlled trial

**DOI:** 10.1186/1745-6215-13-55

**Published:** 2012-05-06

**Authors:** Francesco Burzotta, Marta Francesca Brancati, Carlo Trani, Italo Porto, Antonella Tommasino, Gianluigi De Maria, Giampaolo Niccoli, Antonio Maria Leone, Valentina Coluccia, Giovanni Schiavoni, Filippo Crea

**Affiliations:** 1 Institute of Cardiology, Catholic University of the Sacred Heart, L.go Gemelli 8, 00168, Rome, Italy

**Keywords:** Bare-metal stent, Drug-eluting balloon, Percutaneous coronary interventions

## Abstract

**Background:**

Neointimal hyperplasia plays a pivotal role in the pathogenesis of in-stent restenosis in patients undergoing percutaneous coronary interventions. Drug-eluting balloons are a promising tool to prevent restenosis after coronary angioplasty. Moreover, an increased knowledge of the pathophysiology of restenosis my help improve therapeutic strategies.

**Methods/Design:**

We present the design of an open-label, randomized three-arm clinical trial aimed to assess whether a strategy of bare-metal stent implantation with additional use of drug-eluting balloons, either before (pre-dilation) or after stenting (post-dilation), reduces the primary endpoint of in-stent neointimal hyperplasia area as compared with a strategy of bare-metal stent implantation alone. This primary endpoint will be assessed by optical coherence tomography at follow-up. Secondary endpoints will be the percentage of uncovered struts, and the percentage of struts with incomplete apposition. An ancillary study investigating the relation between systemic levels of endothelial progenitors cells and neointimal hyperplasia, and the interaction between endothelial progenitors cell levels and drug-eluting balloons has been planned. Thirty consecutive patients undergoing percutaneous coronary intervention will be randomized with a 1:1:1 design to bare-metal stent implantation alone (n = 10); bare-metal stent implantation after pre-dilation with a drug-eluting balloon (n = 10); or bare-metal stent implantation followed by post-dilation with a drug-eluting balloon (n = 10). Six-month follow-up coronary angiography with optical coherence tomography imaging of the stented segment will be performed in all patients. Blood samples for the assessment of endothelial progenitors cell levels will be collected on admission and at 6 months.

**Discussion:**

Experimental and early clinical data showed that inhibition of neointimal hyperplasia may be obtained by local administration of antiproliferative drugs loaded on the surface of angioplasty balloons. The INtimal hyPerplasia evAluated by oCT in *de novo* COROnary lesions treated by drug-eluting balloon and bare-metal stent (IN-PACT CORO) trial was conceived to test the superiority of a strategy of bare-metal stent implantation with additional drug-eluting balloon use (either before or after stenting) versus a strategy of bare-metal stent implantation alone for the reduction of neointimal hyperplasia. We also planned an ancillary study to assess the role of endothelial progenitors cells in the pathophysiology of neointimal hyperplasia.

**Trial registration:**

Clinicaltrials.gov NCT01057563.

## Background

Restenosis due to neointimal hyperplasia causes repeat target vessel revascularization in a relevant number of patients undergoing percutaneous coronary interventions (PCI).

Drug-eluting stents (DES) are currently widely adopted to reduce the restenosis rate and repeat revascularizations [1]. However, DES technology is associated with a profound inhibition of stent strut endothelialization, which may lead to the presence of uncovered stent struts and to the persistence of polymer inducing inflammatory reactions in the vessel wall [2,3]. Such factors may increase the risk of stent thrombosis so that prolonged dual antiplatelet therapy is recommended after DES implantation [4].

Experimental data [5] and early clinical experiences showed that inhibition of neointimal hyperplasia may be obtained by local administration of antiproliferative drugs (like paclitaxel) loaded on the surface of angioplasty balloons [6-8]. Accordingly, drug-eluting balloons (DEBs) are a promising tool to prevent restenosis and avoid the undesirable persistence of DES polymers in the vessel wall, thus potentially increasing the safety of PCI. Most of the scientific evidence regarding DEB efficacy is actually concentrated in the treatment of patients with in-stent restenosis; there are some data about use of DEBs for *de novo* lesions and for bifurcation lesions [9]. For *de novo* lesions the only registry available is the Paclitaxel-Eluting PTCA-Balloon Catheter to Treat Small Vessel Coronary Artery Disease (PEPCAD I), which concluded that DEBs are associated with a high procedural success rate in small *de novo* lesions, while DEBs in conjunction with a bare-metal stent (BMS) remains a concern because of high restenosis rate [10]. Until now, DEBs failed to show equivalence to DES regarding angiographic endpoints during PCI of small coronary arteries [11].

Optical coherence tomography (OCT) is a novel imaging modality with high resolution to assess neointimal coverage and stent strut apposition [12-14]. It was used in a recent study [15] evaluating the effect of sequential application of DEBs and BMS (for *de novo* coronary lesions) on neointimal hyperplasia; the sequence of application (DEB first versus BMS first) did not influence outcome, except for better apposition when applying the BMS first. However, in this study there was no control group (that is, BMS alone).

Thus, we designed an open-label, single center, randomized trial to evaluate whether additional DEB use in patients undergoing BMS implantation, either before or after the BMS implantation, improves neointimal formation assessed by OCT and affects the process of strut coverage at follow-up, compared with BMS implantation alone.

Moreover, recent clinical observations have suggested that endothelial progenitor cells (EPC), may play a role in the process of in-stent restenosis [16,17]. We therefore planned an ancillary study, aiming at assessing the relation between levels of systemic EPC and neointimal hyperplasia at follow-up.

### Aims of the main study

To assess whether a strategy of BMS implantation with additional DEB use, either at the time of pre-dilation or of post-dilation:

1. reduces neointimal hyperplasia, as compared to a strategy of BMS implantation alone;

2. affects strut coverage and strut malapposition.

### Aims of endothelial progenitor cell ancillary study

To evaluate possible correlations of EPC levels with neointimal hyperplasia, stent coverage and stent malapposition.

## Methods/Design

### Study design

The INtimal hyPerplasia evAluated by oCT in de novo COROnary lesions treated by drug-eluting balloon and bare-metal stent (IN-PACT CORO) trial is a single center, open-label, randomized trial enrolling 30 consecutive patients undergoing PCI with BMS implantation. Recruited patients will be randomized 1:1:1 to three arms:

1. BMS implantation (BMS group).

2. BMS implantation after lesion pre-dilation with DEB (PRE-DEB group).

3. BMS implantation followed by post-dilation with DEB (POST-DEB group).

Clinical follow-up will be performed at 1, 6 and 12 months. Enrolled patients will undergo a 6-month follow-up coronary angiography with OCT evaluation of the stented segment using the C7-XR^TM^ Coronary Imaging System (LightLab Imaging Inc., Westford, MA, USA). OCT analysis will be performed off-line by expert OCT analysts blinded to the treatment assignment.

The study protocol was conceived in March 2009, conformed to the Declaration of Helsinki, and was approved by the Ethical Committee of our center. We prepared a written informed consent which patients will be asked to sign to be enrolled in the protocol.

**Figure 1:**
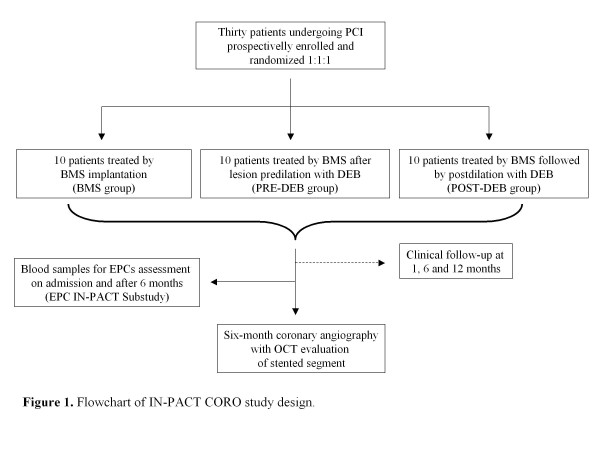
The study flow-chart.

The full study flow-chart is represented in Figure 1.

### Study endpoints

The primary endpoint is 6-month in-stent neointimal hyperplasia area assessed by OCT.

Secondary endpoints are the 6-month percentage of uncovered struts and the 6-month percentage of struts with incomplete stent apposition.

### Eligibility, inclusion and exclusion criteria

Eligible patients have to be at least 18 years old; both genders are eligible but women with child-bearing potential are not accepted.

Since the study will recruit a small number of patients, we selected a homogeneous population of stable non-diabetic patients undergoing elective PCI with BMS and already on statin at target dose for low-density lipoprotein cholesterol level <100 mg/dL. Since diabetes mellitus is known to dramatically increase neointimal hyperplasia after BMS implantation (primary endpoint of this study) and acute coronary syndromes may influence acute stent apposition to the vessel wall (one of the secondary endpoints), we decided to exclude such clinical conditions in order to reduce the possibility of any imbalance of confounding factors.

We selected *de novo*, non-complex lesions with length ≥10 mm and ≤ 25 mm, located in straight segments of vessels whose size requires a single stent with diameter of 3.0 to 3.5 mm. Clinical and angiographic inclusion criteria are summarized in Table 1.

**Table 1:** Inclusion criteria

**Clinical inclusion criteria**	Non-diabetic patients with a stable coronary artery disease, undergoing elective PCI with BMS
	patients already on statin, at target cholesterol level (low-density lipoprotein cholesterol <100 mg/dL)
**Angiographic inclusion criteria**	*De novo* non-complex lesions located in straight coronary segments
	lesion length ≥10 mm and ≤25 mm
	vessel size requiring a single stent with diameter between 3.0 and 3.5 mm

#### Clinical exclusion criteria

Clinical exclusion criteria are:

age <18 years or impossibility to give informed consent;

women with child-bearing potential;

diabetes mellitus;

life expectancy less than 6 months or any condition impeding clinical follow-up (for example, no fixed address);

significant platelet count alteration (<100,000 cells/mm^3^ or >700,000 cells/mm^3^);

gastrointestinal bleeding requiring surgery or blood transfusions within the previous 4 weeks;

participation to another study with any investigational device or drug which is still in the active phase;

infective, neoplastic or autoimmune diseases;

history of clotting pathology, known hypersensitivity to aspirin, heparin, cobalt- chromium, paclitaxel or contrast dye;

renal failure with creatinine value >2.5 mg/dL;

poor cardiac function as defined by left ventricular global ejection fraction ≤30%;

acute myocardial infarction within the past 48 hours;

non ST-elevation acute coronary syndrome within the past 48 hours.

#### Angiographic exclusion criteria

Angiographic exclusion criteria are:

left main coronary artery disease;

lesions in coronary artery bypass grafts;

coronary anatomy not suitable for OCT scan;

bifurcation lesions, chronic total occlusions, severe calcifications or moderate-to-severe tortuosities;

presence of additional non-target lesions requiring treatment, within and outside the target vessel, which are not successfully treated (non-target lesions should be treated prior to the target lesion).

### Technical details of the percutaneous coronary interventions procedure

#### General considerations on drug-eluting balloon usage

A DEB is mainly intended to serve as drug delivery to the vessel wall and should therefore always cover the stenotic area as well as adjacent vessel segments covered by a stent or dilated by a balloon catheter, incidentally or by intention.

DEB length and positioning within the target lesion will therefore be carefully chosen to avoid geographic miss between DEB and treated vessel segments.

#### Characteristics of the drug-eluting balloon used in the study

IN.PACT^TM^ Falcon^TM^ is a paclitaxel-eluting PCI balloon catheter manufactured by Invatec Technology Center GmbH (Hungerbüelstrasse 12, 8500 Frauenfeld – Switzerland). Model mix includes Rapid Exchange (RX) and, for some sizes, Over The Wire (OTW) design. The balloon is coated with FreePac^TM^, a paclitaxel-eluting formulation, at the dose of 3.0 μg per mm^2^ of the balloon surface.

The IN.PACT^TM^ Falcon^TM^ RX dilatation balloon catheter is composed of a proximal single-lumen shaft, a dual-lumen distal shaft and a balloon close to the catheter tip. The proximal shaft consists of a stainless steel hypotube with a proximal end luer-lock connector (hub) for balloon inflation. At the opposite side, a special transition construction guarantees an optimal push-torque transmission through the full catheter length. The first lumen of the distal shaft is dedicated to guide wire passage while the other, which continues through the proximal shaft up to the hub, is dedicated to the inflation of the balloon. Maximum guide wire diameter is 0.014 inches (0.36 mm). A flushing needle with a luer port is provided in the sterile packaging to facilitate the flushing of the guide wire lumen prior to usage.

The IN.PACT^TM^ Falcon^TM^ OTW dilatation catheter consists of a dual-lumen shaft ending proximally with a Y connector (hub) and distally with a balloon closed to the catheter tip. The straight port of the Y connector is the guide wire entrance and the side port is used to inflate and deflate the balloon. Both lumens run through the entire shaft length. The guide wire lumen permits the use of guide wires to facilitate advancement of the catheter to and through the stenosis to be dilated and it ends at the tip of the catheter. Maximum guide wire diameter is 0.014 inches (0.36 mm).

The balloon is designed to reach specific diameters at specific pressures. A single central radiopaque marker and/or two radiopaque markers are available in order to correctly position the balloon under fluoroscopy. IN.PACT^TM^ FalconTM is available in different balloon sizes.

The majority of the drug is released within the first 30 seconds of balloon inflation. The duration of the inflation should therefore be between 30 seconds and 1 minute for optimal drug release.

### Percutaneous coronary interventions procedure description according to randomization

1. BMS group procedure:

Lesion pre-dilation with an undersized semi-compliant balloon (balloon to artery ratio, 0.5:1).

BMS implantation (stent to artery ratio, 1.1:1).

Post-dilation of the stented segment with a non-compliant balloon at high pressure (16 to 18 atm).

2. PRE-DEB group procedure:

a. Pre-dilation

Pre-dilation of the target lesion with an undersized semi-compliant standard percutaneous transluminal coronary angioplasty (PTCA) balloon (balloon to artery ratio, 0.5:1).

b. DEB dilation

DEB diameter and pressure: nominal DEB diameter must be chosen to guarantee full vessel wall contact at a pressure close to or slightly higher than the DEB nominal pressure (balloon to artery ratio, 1:1).

DEB length: nominal DEB length must exceed 10 mm (5 mm per edge), the length of the stent which is planned to be deployed.

DEB inflation time: 45 seconds.

c. BMS implantation

d. Post-dilation

Post-dilation of the stented segment must be performed with a non-compliant PTCA balloon.

Balloon diameter: nominal PTCA balloon diameter must be chosen to reach a balloon to stent ratio of 1:1 at high pressure (16 to 18 atm).

Balloon length and positioning: PTCA balloon length should be shorter than the length of the deployed stent. In case of post-stent edge residual stenosis, post-dilation balloon must fall within are outside the stent (5 mm per edge) which was the previously dilated by the DEB.

3. POST-DEB group procedure:

a. Pre-dilation

Pre-dilation of the target lesion must be performed with an undersized semi-compliant standard PTCA balloon (balloon to artery ratio, 0.5:1).

b. BMS implantation

Stent to artery ratio, 1.1:1.

Stent length must allow full coverage of the target lesion with a single stent as well as be 10 mm shorter than the DEB which the operator is planning to use next.

c. Post-dilation

Post-dilation of the stented segment must be performed with a non-compliant PTCA balloon.

Balloon diameter: nominal PTCA balloon diameter must be chosen to reach a balloon to stent ratio of 1:1 at high pressure (16 to 18 atm).

d. DEB dilation

DEB length and positioning: DEB length must be 10 mm longer than the previously deployed stent (or than the extended pre-treated area in case of former post-dilation outside the stent edges) and centered within such pre-treated length (5 mm per edge).

DEB inflation time: 45 seconds.

Balloon to stent ratio: 1.1:1 at a pressure close or slightly higher of the DEB nominal pressure.

The angiographic results of the procedure will be assessed by three-dimensional quantitative coronary angiography (QCA) using the Paieon Cardi-Op system (Paieon, Inc., 747 Third Avenue, New York 10017-2803, United States).

### Post-procedural management

Cardiac damage markers (creatine-kinase-MB and troponin I) will be assessed before the procedure, and 6 hours and 24 hours after PCI. Thereafter, further blood samples will be performed only if clinically indicated.

After PCI, patients will be given aspirin (75 to 100 mg/d) and life-long clopidogrel (75 mg/d) for ≥3 months (according to the on-label prescription for DEB-treated patients).

### Follow-up

Clinical follow-up will take place at 1 month (±1 week), 6 months (±2 weeks) and 1 year (±30 days) by clinical visit or phone interview.

At the 6-month (± 2 weeks) follow-up, all patients will undergo a coronary angiography (with three-dimensional QCA) and an OCT study.

We anticipate a patient drop-out rate of 10%.

### Rationale for optical coherence tomography selection and description of optical coherence tomography analysis

For many years, QCA has been used to assess regression and progression of coronary obstructions in pharmacological interventions, to assess the efficacy of PCI and stenting, and for vessel sizing. However, QCA late lumen loss is the difference between two minimal lumen diameter measurements at two different times, and the axial location of this diameter is variable at each time point. An angiogram only examines the lumen, while the disease is in the vessel wall. These limitations have spurred the search for new intravascular diagnostic imaging techniques. A meta-analysis of QCA versus intravascular ultrasound (IVUS) parameters for assessing stent restenosis [18] showed by regression analysis that QCA late lumen loss and percentage of diameter stenosis correlates only moderately with IVUS evaluation of neointimal hyperplasia. A recent work [19] evaluated the correlation of angiographic late loss with the degree of in-stent neointimal proliferation assessed by OCT, demonstrating a poor correlation of angiographic late loss with OCT at low degrees of neointimal proliferation (R = 0.38).

The detection of the post-stent implantation intima by OCT and IVUS has also been compared. After a median follow-up time of 6 months, Matsumoto *et al*. examined the reaction of the intima by OCT and found that 64% of the struts had become covered with an intima <100 μm thick (below the resolution of IVUS) [20]. The correlation between OCT and histology measurements (r = 0.980, *P* <0.001 for lumen area; r = 0.978, *P* <0.001 for stent area; and r = 0.961, *P* <0.001 for neointimal area) was stronger than the correlation between IVUS and histology (r = 0.803, *P* <0.001 for lumen area; r = 0.817, *P* <0.001 for stent area; and r = 0.776, *P* <0.001 for neointimal area). In addition, the diagnostic accuracy for detecting a small degree of neointimal proliferation by OCT (area under the curve = 0.967, 95% confidence interval 0.914 to 1.019) was higher than that by IVUS (area under the curve = 0.781, 95% confidence interval 0.621 to 838) [21].

The frequency of detection of stent malapposition is greater with OCT than with IVUS. In a recent *in vivo* study by Kubo *et al*., the detection rate with OCT was 47% in a sample of 55 patients, while with IVUS it was only 18% (*P* <0.001) [22]. Similar results were reported by Bouma *et al*. five years earlier in a study involving experimental animals [23].

So OCT represents a useful clinical tool to study endothelialization of stents and abnormal tissue responses and to detect the presence of delayed healing or incomplete apposition of stents to the arterial wall as a possible mechanism of in stent thrombosis [24,25].

In our study, OCT will be performed with the imaging system C7 XR (LightLab Imaging Inc.), using a non-occlusive technique, with automated intracoronary injection of iso-osmolar contrast. This Fourier domain-OCT system achieves 10 times higher resolution than IVUS, approximately 15 microns, with pullback speed of 2 cm/sec, and an acquisition frame rate of 100 frames/sec.

The entire stent length will be assessed and cross-sectional images will be analyzed every 0.4 mm.

The struts will be classified as uncovered if a tissue layer on the endoluminal surface is not visible or covered in the presence of visible tissue between the endoluminal surface and the lumen.

The tissue coverage thickness will be measured in each strut as the distance from the strut endoluminal surface to the lumen. In each cross-section analyzed, a series of parameters will be calculated.

### Endpoint assessment

The primary endpoint is in-stent neointimal hyperplasia area, that is stent area minus lumen area [26] and its percentage (tissue coverage area/stent area × 100) evaluated by OCT. The other related parameters of tissue coverage thickness (μm), tissue volume coverage (tissue coverage area × stent length) and its percentage (tissue coverage volume/stent volume × 100) will also be evaluated. To assess the pattern of coverage, the ratio between the difference of maximum and the minimum tissue thickness coverage (minimum tissue thickness coverage/maximum tissue thickness coverage) will be calculated in each frame. A ratio close to 1 indicates an asymmetric tissue coverage, on the opposite a ratio close to 0 indicates a symmetric tissue coverage.

The secondary OCT endpoints are described in Table 2. Incomplete strut apposition (ISA) will be defined as a distance between strut endoluminal surface and the vessel wall higher than strut thickness. ISA will be considered present if at least one single strut is incompletely apposed to the vessel wall. In each OCT frame analyzed, the number of struts with ISA and the maximum distance from the endoluminal stent strut to the vessel wall will be measured.

**Table 2:** Secondary endpoints

**Percentage of uncovered struts**	Number of uncovered struts/total number of struts
**Percentage of struts with incomplete strut apposition (ISA)**	Number of struts with ISA/total number of struts

### Sample size calculation and statistical analysis

The IN-PACT CORO trial is open-label, randomized clinical trial in which patients will be randomly assigned, with a 1:1:1 design, to BMS implantation alone, BMS implantation with pre-DEB use, and BMS implantation with post-DEB use. The primary aim is to test the superiority of a strategy of BMS implantation with pre- or post-DEB use versus a strategy of BMS implantation alone on the primary endpoint of in-stent neointimal hyperplasia area, assessed by OCT at follow-up. Secondary endpoints will be the percentage of uncovered struts, and the percentage of struts with ISA.

Little information is available on neointimal proliferation after BMS implantation: two small non-randomized studies [13,27] reported maximal and minimal neointimal thickness (mm) at 7.3 month follow-up (first study) and mean neointimal thickness at 8 month follow-up (latter study) being more than four-fold higher in the BMS group compared with the sirolimus (rapamycin)-eluting stent group, although data on neointimal area are not available. In the Harmonizing Outcomes With Revascularization and Stents in Acute Myocardial Infarction (HORIZONS-AMI) OCT sub-study, a mean neointimal area of 2.8 ± 1.4 was reported 13 months after a BMS implantation [28].

A recent randomized study comparing 12 polymer-coated rapamycin-eluting stents to 12 non- polymer rapamycin-eluting stents [29] reported a neointimal area of 0.3 ± 0.2 mm^2^ in the polymer stent versus 1.2 ± 0.8 mm^2^ in the non-polymer stent, with a difference of 0.9 mm^2^ (95% confidence interval, 0.3 to 1.4).

We have hypothesized that additional DEB use (either before or after BMS implantation) will yield a neointimal area similar to that reported in the non-polymer rapamycin-eluting stent (that is, 1.2 mm^2^) and that this value corresponds to an approximately 50% reduction of mean neointimal area in the group receiving BMS implantation alone (hypothesizing a control group hyperplasia better than that reported in the BMS arm of the HORIZONS-AMI OCT sub-study). As two co-primary endpoints are pre-specified: that is, either pre- or post-DEB use reduces neointimal hyperplasia as compared to BMS alone, a type I error of 2.5% will be allocated to each endpoint to preserve the total type I error at the 5% level. Power is set to 85% for each primary endpoint. To detect such difference, 10 patients will be required in each group. Since we anticipate a drop-out rate of 10%, we will recruit another patient for each group in case of loss to follow-up or unanalyzable studies.

Continuous variables will be reported as mean and standard deviation or as median and interquartile range (according to their distribution) and comparisons between two groups will be made with unpaired *t-*test or Mann Whitney *U-*test, as appropriate. Categorical variables will be presented as numbers and frequencies and comparisons will be made using Chi-square test or Fisher’s exact test, as appropriate.

### Endothelial progenitor cell ancillary study design

A correlation analysis will be performed to ascertain whether EPC contribute to neointimal regrowth in such patients.

Peripheral blood samples of all patients enrolled in the IN-PACT CORO protocol will be obtained on admission and after 6 months of follow-up (Figure 1).

### Endothelial progenitor cells levels assessment for endothelial progenitor cells ancillary study

The cytofluorimetric assays will be performed on admission and after 6 months’ follow-up. Peripheral blood will be drawn and buffered using sodium citrate. One hundred microliters of blood will be incubated with 5 μL of phycoerythrin-conjugated monoclonal antibody against CD34, with 5 μL of fluorescein isothiocyanate-conjugated monoclonal antibody against kinase insert domain receptor (KDR) and with 5 μL of monoclonal antibody against CD45. Fluorescence-activated cell sorting assay will be performed according to the specific log gain of forward and side scatter. EPC will be detected as cells CD34+ KDR + CD45- The negativity for CD45 is fundamental to distinguish EPC from other nucleated cells in peripheral blood, represented by leukocytes [30].

### Study limitations

The major anticipated limitation of the present study is that it is an open-label and not a double-blinded trial. In order to increase the feasibility of this spontaneous, non-sponsored study, we decided to perform an open-label trial. The treating physicians cannot be blinded since the DEB device is fairly different from a standard balloon due to its thick white covering material.

However, OCT analyses will be performed by an expert OCT image analyzer who is not involved in patient recruitment and blinded to patient randomization and clinical status.

## Trial status

Patient recruitment is ongoing.

## Abbreviations

BMS, Bare-metal stent; DEB, Drug-eluting balloon; DES, Drug-eluting stent; EPC, Endothelial progenitor cells; ISA, Incomplete strut apposition; IVUS, Intravascular ultrasound; KDR, Kinase insert domain receptor; OCT, Optical coherence tomography; OTW, Over The Wire; PCI, Percutaneous coronary intervention; POST-DEB, Post-dilation with drug-eluting balloon; PRE-DEB, Pre-dilation with drug-eluting balloon; PTCA, Percutaneous transluminal coronary angioplasty; QCA, Quantitative coronary angiography; RX, Rapid Exchange.

## Competing interests

The authors declare that they have no competing interests.

## Authors’ contributions

FB conceived the main study and drafted the manuscript. CT made substantial contribution in study design and coordination. IP conceived the EPC ancillary study and helped to draft the manuscript. MFB helped to draft the manuscript and participated in study coordination and acquisition of data. AT, GN and AML revised the manuscript with important intellectual contribution. GDM and VC participated in acquisition of data. GS and FC have given final approval of the version to be published. All authors read and approved the final manuscript.

## Funding

The authors declare that there is no funding source for this study.
